# Patient demands for ethnic–based separation in public hospitals in Israel: patients’ and practitioners’ perspectives

**DOI:** 10.1186/s13584-018-0237-9

**Published:** 2018-11-15

**Authors:** Yael Keshet, Ariela Popper-Giveon

**Affiliations:** 1 Western Galilee Academic College, P.O. Box 2125, 24121 Akko, Israel; 2 David Yellin Academic College, POB 3578, 91035 Jerusalem, Israel

**Keywords:** Ethnic segregation, Inpatient rooms, Hospitals, Policy, Israel, Arabs

## Abstract

**Background:**

Segregation within the healthcare system is commonly associated with disparities in the utilization of health services and in the outcomes of medical care. In Israel, the Jewish majority and the Arab minority populations are treated in the same healthcare organizations. Nevertheless, demands for ethnic separation in inpatient rooms are raised at times by patients, despite the principle of prohibiting discrimination between patients on the grounds of religion, race, sex and nationality. The study sought to examine patients’ attitudes regarding separation between Jews and Arabs in inpatients rooms, and to discover the coping strategies employed by healthcare practitioners.

**Methods:**

A mixed methodology was employed. We conducted a survey of a representative sample of the Israeli population (*N* = 760); and held 50 in-depth interviews with nurses, physicians and managers employed in 11 public hospitals in Israel.

**Results:**

In the representative sample survey, 30% of Jews and 21% of Arabs agree that patients should be allowed to choose to be placed in an inpatient room in which only patients of their own ethnic group are hospitalized. Among both Jews and Arabs, a high level of religiosity and a low level of education predict this position. Most Jews (80%) and Arabs (71%) do not agree that the entire healthcare system should be ethnically separated.

The in-depth interviews revealed evidence of demands for ethnic separation made at times by Jewish patients, which are often met by the nurses. In some cases, nurses separate Jewish and Arab patients of their own accord. They do this either to promote cultural compatibility between patients or to avoid unnecessary tension and confrontations. In some cases, this step may constitute discrimination against Arab patients. Managers and senior physicians, for their part, are generally unaware of this ethnic separation, or deny that it takes place.

**Conclusions:**

Ethnic separation in inpatient rooms does take place some of the time and this runs contrary to the ethos of neutrality in medicine. We recommend implementation of a specific national policy that prohibits ethnic-based separation in hospitals’ inpatient rooms. Better communication is required to ensure that policy decisions are clearly conveyed to the wards and that segregation does not become institutionalized.

## Background

This article addresses the phenomenon of ethnic-based separation of some patients in inpatient rooms in Israeli public hospitals. This issue has significant ethical and practical implications, which ought to be addressed by policymakers in the healthcare system. The study employed a mixed quantitative and qualitative method, through which we examined patients’ attitudes toward separation between Jews and Arabs in inpatient rooms, as well as the coping strategies adopted by the staff in face of demands for separation.

The Israeli public healthcare system provides an excellent setting in which to research this topic. It employs Jewish and Arab practitioners who work side by side treating both Jewish and Arab patients in the context of a prolonged and violent conflict between Israel and the Palestinians. The Israeli-Palestinian conflict, the tension between the Jewish majority and the Arab minority^1^ populations within Israel, and the high number of Arab practitioners employed in Israel’s public healthcare system all have a strong bearing on patients’ preferences, as well as on practitioners’ attitudes and coping strategies.

Although much of the research literature regarding segregation in healthcare systems focuses on segregation *between* hospitals and *between* health care providers, the present article turns the spotlight onto ethnic-based separation *within* hospitals – in the inpatient rooms.

### Segregation in the healthcare system

The international literature indicates that segregation in healthcare systems is commonly associated with disparities in the utilization of health services and in the outcomes of medical care. An uneven distribution of facilities and services available to different groups may yield disparities in health; for example, if hospitals that serve minorities are under-funded and the availability of specialized services within them is limited, then the medical treatment they offer will tend to be inferior [26]. Ethnic segregation in the healthcare system may take the form of an unequal distribution of patients by ethnicity across hospitals. This occurs when racial or ethnic groups are distributed unevenly across neighborhoods or regions, and is referred to in the literature as residential segregation [10].

Factors beyond residence, such as income, may also play a significant role in determining where and how healthcare services are sought and obtained. In the United States, extensive research literature has documented racial and ethnic inequalities in the use of health services and in the outcomes of medical care [5]. While overt segregation in hospitals was essentially eliminated during the 1960s, de facto segregation remains in effect, partly owing to social and economic pressures that are unique to the healthcare system. Apart from residential segregation and socioeconomic status, factors that may result in racially segregated hospitals include racial differences in physician referrals, unequal access to transportation systems, institutional discrimination, and patient preferences [26].

Segregation in the healthcare system runs contrary to the principle of neutrality in medicine, which guarantees apolitical, impartial and humanitarian healthcare. All health services and personnel are expected to adhere to this principle, which in practice means that health practitioners must provide services “on the basis of need alone, giving priority to the most urgent cases of distress and making no distinctions on the basis of nationality, race, gender, religious belief, class or political opinions” [15].

#### The Israeli context

Arabs form the largest ethnic minority in Israel, comprising about 21% of the population. Arabs in Israel have full citizenship and by law Jewish and Arabs citizens have equal rights and entitlements. However, Arabs still constitute a minority that suffers from discrimination and disadvantages in income, education and employment [17]. Moreover, the prolonged and violent Israeli-Palestinian conflict has generated high levels of mistrust and social tension between the two ethno-national groups that coexist in the State of Israel [23]. In the context of the Israeli-Palestinian conflict, the Arab citizens of Israel are divided in their loyalty to the state of Israel and their Palestinian kin. At the same time, many Jews perceive them as a hostile fifth column and regard them with suspicion and aversion [2].

Arabs and Jews in Israel are highly segregated in terms of where they live and where children attend school. The Arab population is concentrated in three geo-cultural areas: the Galilee (northern Israel), the “Little Triangle” (in Israel’s center), and the Negev (in the south). Most Arabs live in distinct Arab localities, and only 15% live in mixed Jewish-Arab cities [24]. Although both Jewish and Arab children in Israel study in schools financed and supervised by the Israeli Ministry of Education, they attend separate schools. Only some 6% of all the pupils in Israel attend schools in which Arab and Jewish pupils sometimes encounter one another [25]. Many Arab children do not meet Jews until they reach university or enter the labor market. Throughout the 1990s, although, greater access to higher education for Arabs has reduced the educational and income gaps between Jews and Arabs in Israel [1, 28]. Many Arab students currently study in all of Israel’s academic institutions, although Arabs are still substantially under-represented in higher education. While Arabs constitute 21% of the Israeli population, and about 26% of the relevant age group, in the 2017 academic year, they constitute 15% of students enrolled in academic institutions [6].

Public healthcare organizations in Israel, on the other hand, in which Jewish practitioners treat Arab patients and vice versa, are perceived as an apolitical neutral space, as a unique enclave in Israeli society, where Jews and Arabs meet and integrate. Arabs constitute a large and increasing proportion of Israeli nurses, pharmacists and physicians [13, 18–20]. Both the Jewish majority population and the Arab minority population use the same healthcare facilities for secondary and tertiary care. There is no policy of ethnic-based segregation in Israeli hospitals. Jewish and Arab patients are admitted to the same hospitals, share the same inpatient wards and often share the same rooms, where Jewish and Arab visitors, the patients’ friends and relatives, also intermingle.

Nevertheless, despite the perception of public hospitals in Israel as a neutral space free of political conflict, stories and reports of ethnic-based separation between Jewish and Arab patients appear in the media from time to time [8, 16]. According to these stories, the principal driver of this phenomenon are the demands of some Jewish patients to be separated from the Arab patients and to be treated in separate rooms, together with other Jewish patients. This has been most prominent in the case of maternity wards.

In 2016, a member of the Israeli parliament named Bezalel Smotrich, of the (right wing) Jewish Home party, tweeted his support for the separation of Arab and Jewish mothers in impatient rooms within maternity wards in Israeli public hospitals. This tweet responded to a report on Israeli Radio that revealed that some hospitals regularly agreed to separate Arab and Jewish mothers in maternity wards when the Jewish mothers request this. Smotrich’s tweet generated substantial criticism in the public, the media, and in political circles. In the wake of this criticism, Smotrich went a step further, declaring that “It’s natural that my wife wouldn’t want to lie [in a bed] next to a woman who has just given birth to a baby who might want to murder my baby twenty years from now” [8].

In Israel, the prohibition of ethnic separation in hospital inpatient rooms is implied in the patient’s rights law, according to which “a caregiver or medical institution shall not discriminate between patients on the grounds of religion, race, sex, nationality, place of origin etc.” There is, therefore, a law in Israel that clearly prohibits discrimination, but there is no specific policy prohibiting separation among patients in hospital impatient rooms, since this is sometimes perceived as a step toward cultural competency. According to a report by the Ministry of Health [11], following the exposure of this issue in the media, the Ministry of Health issued a statement that rejected this practice, and summoned hospitals’ directors to make this clear to them.

It is difficult to ascertain the motivation of ethnic separation in hospital inpatient rooms; namely, to distinguish between separation that stems from a desire to promote cultural competency and separation that stems from discrimination and racism [11]. The report [11] declares that respecting cultural diversity does not condone separation of patients or preferential treatment of one group, but is intended to promote “fairness” in the health services. However, alongside the progress that has been made, this approach may generate problems, for example, when “cultural competency” or “patient experience” become a fig leaf for racial or discriminatory differentiation.

Although the phenomenon of separating certain patients in public hospitals in Israel according to their ethnic identity surfaced in the media and in popular culture, we have found no study that addresses its prevalence and its causes. Research into the experiences and attitudes of practitioners and patients, both Jews and Arabs, regarding ethnic-based separation of Jewish and Arab patients within public hospitals will help to formulate clear guidelines on the subject and to offer practitioners and managers the information needed to develop effective tools to handle these situations.

## Methods

The present article reports on a study that employed two complementary methods, quantitative and qualitative. The quantitative method was used to examine the attitudes of the Israeli population toward ethnic-based separation within the healthcare system. Using the qualitative method, we studied the points of view of Jewish and Arab managers, physicians and nurses employed in Israeli public hospitals concerning this phenomenon.

### Quantitative study

A questionnaire was constructed by adapting the questions that comprised previous questionnaires [3, 21, 27] to the objectives of the present research and to the Israeli context. The questionnaire included seven demographic questions, five questions pertaining to treatment preference and refusal, and 16 statements regarding attitudes and sentiments concerning ethnicity in healthcare. For the purposes of this article we used responses to two statements that examined attitudes regarding placement in separate rooms and attitudes regarding separate healthcare facilities. The other statements pertain to preferences for a certain physician or refusal of treatment based on his ethnicity and are detailed elsewhere (Authors, submitted). The respondents’ statements were rated on a Likert scale between 1 – absolutely disagree, and 5 - fully agree. The questionnaire was formulated in Hebrew and translated (and translated back) into Arabic and Russian, thereby covering the three most commonly used languages in Israel.

Structured telephone interviews were conducted between December 2016 and January 2017 by an experienced survey company (Unisker – University of Haifa) hired by the researchers. Random sampling within stratified Jewish and Arab subgroups was performed to obtain a representative sample of Jews (*n* = 505) and Arabs (*n* = 255) (out of a gross sample of 1355) living in Israel at the time of the survey, aged 18 years or older. Oral informed consent was obtained prior to commencing the interview. Interviews were conducted by experienced interviewers, fluent speakers of Hebrew, Arabic or Russian, depending on the interviewee’s native language. The entire questionnaire was completed during a single phone call, and the respondent’s anonymity was maintained.

The survey data were analyzed using SAS software, version 9.4 (SAS Institute Inc.). Standard descriptive statistics were used to characterize the sample. Associations between variables were evaluated using the Kruskal-Wallis test (continuous variables) or the chi-square test (categorical variables), as appropriate. A multiple logistic regression analysis was run to determine the odds ratio (OR) and 95% confidence interval (CI) of factors predictive of attitudes regarding placement in separate rooms. *P* values smaller than 0.05 were considered statistically significant.

### Qualitative study

We used qualitative research methods to learn about individual healthcare practitioners’ points of view, experiences and coping strategies regarding ethnic-based separation within public hospital wards in Israel.

Fifty in-depth interviews were conducted during 2016–2017 with ten managers (senior nurses, department heads, a hospital director, etc.) (3 Arabs and 7 Jews) and with forty Jewish and Arab healthcare professionals—physicians (10 Arabs and 10 Jews) and nurses (10 Arabs and 10 Jews) —employed at eleven public hospitals in Israel (aged 26–74, mean age 43). A snowball sampling technique was adopted to recruit participants. This method is employed extensively in studies dealing with sensitive matters [14]. We chose to use it since we preferred not to approach individual practitioners through their workplace. Researchers who use the snowball form of sampling initially select a small number of participants and ask them to recommend others who meet the designated criteria and may agree to participate in the study. While this form of recruitment could limit heterogeneity among participants, we made a point of interviewing practitioners who worked in a wide range of large hospitals located in various parts of the country in order to expand the variance of the sample.

The 30 to 90-min interviews were conducted in Hebrew, a language all participants speak fluently. After securing permission from participants, interviews were tape recorded, then transcribed verbatim and analyzed using Atlas.ti v7.5.17 textual analysis software for systematic coding and inductive analysis. The research was financed by the Israel National Institute for Health Policy Research and approved by the ethics committee of the Western Galilee Academic College.

## Results

### Quantitative results - attitudes among the population

#### The survey population

In total, 760 interviews were completed, out of a gross sample of 1355. Thus, the response rate was 56%. The reasons for non-participation were refusal to answer (341); abandoning the attempt to make a connection owing to multiple rejections (144); the respondent had trouble answering (79); the interview was terminated before completion (31).

The demographics of the present survey respondents are broadly similar to those of the general Israeli Jewish population as found by ICSB [12]. The survey’s Arab respondents resemble the distribution of the Israeli Arab population with regard to religion. However, among the Jewish respondents there were more adults and fewer young people than in the Jewish Israeli population. The level of education among Jewish respondents is lower than that in the overall Jewish population. These two trends also appear among the Arabs, but because this is a smaller group of respondents these differences between the distribution in the group of respondents and in the general population are less marked. Among the Arab respondents, the self-reported health condition is better than that among the Arab population (Table 1).

**Table 1:** The survey respondents compared to the Israeli population

		Jews			Arabs		
		Survey respondents	Israeli population (ICBS, 2016)	P (Chi-Square Test)	Survey respondents	Israeli population (ICBS, 2016)	P (Chi-Square Test)
		N (%)	%		N (%)	%	
Religion	Jewish	499 (100%)	100%				
	Muslim				192 (78%)	77%	0.7324
	Cristian				29 (12%)	14%	
	Druze				22 (9%)	8%	
	Other				3 (1%)	1%	
Gender	Men	229 (45%)	48%	0.2327	116 (45%)	48%	0.4224
	Women	276 (55%)	52%		139 (55%)	52%	
Age structure	18-25	39 (8%)	10%	<.0001	41 (16%)	15%	0.1257
	26-40	89 (18%)	30%		71 (28%)	35%	
	41-65	230 (46%)	40%		118 (46%)	41%	
	66+	142 (28%)	19%		24 (9%)	9%	
Years of schooling	0-12	164 (34%)	27%	0.0022	154 (61%)	57%	0.1787
	13-15	126 (26%)	31%		51 (20%)	25%	
	16-17	199 (41%)	42%		48 (19%)	19%	
Health condition (Self-reported)	Good	430 (87%)	86%	0.8839	226 (90%)	75%	<.0001
	Not good	63 (13%)	14%		25 (10%)	25%	

#### Attitudes toward the separation of patients

Thirty percent of Jewish respondents agreed or strongly agreed that Jewish patients should be allowed to choose to be placed in an all-Jewish inpatient room, compared to 21% of Arab respondents who agreed to the same statement as applied to Arabs (Table 2). Thus, the proportion of Jews who agreed with the statement that Jewish patients should be allowed to choose to be placed in an inpatient room containing only Jewish patients, was greater than the proportion of Arabs who agreed with the statement that Arab patients should be allowed to choose to be placed in an inpatient room containing only Arab patients.

**Table 2:** Patients’ attitudes regarding separation between Jews and Arabs in the healthcare system

The statement	Respondents	Absolutely disagree				Fully agree	Mean	*P*
		1	2	3	4	5		
Jewish patients should be allowed to choose to be in a room only with Jews	Jews *N* = 503	48%	8%	15%	7%	23%	2.51	<.0001
Arab patients should be allowed to choose to be in a room only with Arabs	Arabs *N* = 255	65%	6%	8%	4%	17%	2.02	
There should be a separate healthcare system in Israel for Jews and for Arabs (similar to the current separation in the education system)	Jews *N* = 496	73%	7%	8%	4%	8%	1.68	n.s.
	Arabs *N* = 254	68%	3%	7%	1%	20%	2.04	

Most of the respondents, both Jewish and Arab, oppose the division of the entire Israeli healthcare system into two separate systems - one for Jews and another for Arabs – along the lines of the way in which the Israeli education system is separated. However, 21% of the Arab respondents and 12% of the Jewish respondents agree or strongly agree that there should be separate healthcare systems for Jews and Arabs in Israel (Table 2).

Using a multiple logistic regression analysis that included all tested demographic variables, we found that level of religiosity and level of education, both among Jews and among Arabs, predict the tendency to agree that patients should be allowed to choose to be placed in an inpatient room in which only patients of their own ethnic group are hospitalized. A high level of religiosity (among Jews OR 2.772, CI 2.171–3.540; among Arabs OR 1.950, CI 1.204–3.160) and a low level of education (among Jews OR 0.937, CI 0.882–0.995; among Arabs OR 0.854, CI 0.769–0.949) predict this position. Gender, age, and self-reported health condition do not predict the attitude that favors ethnic-based separation among patients in an inpatient room. The analysis also indicates that place of residence does not predict this position. It should be noted, however, that in our sample only Jewish participants lived in the West Bank and East Jerusalem, since almost all Arabs in these areas are not Israeli citizens and therefore were not included in the survey (Table 3).

**Table 3:** Demographic characteristics that predict agreement with the statement that Jews and Arabs should be allowed to occupy separate inpatient rooms

Demographic characteristics	Jews		Arabs	
	Odds Ratio Estimates (95% CL)	*P* values	Odds Ratio Estimates (95% CL)	*P* values
Level of religiosity	2.772 (2.171–3.540)	<.0001	1.950 (1.204–3.160)	0.0066
Gender male vs. female	1.049 (0.639–1.590)	0.8197	1.585 (0.852–2.974)	0.1458
Area of residence	0.892 (0.497–1.599)	0.7003	–	
Age	1.006 (0.993–1.019)	0.3466	0.995 (0.973–1.017)	0.6292
Years of schooling	0.937 (0.882–0.995)	0.0330	0.854 (0.796–0.949)	0.0032
Self-reported health condition	0.987 (0.733–1.329)	0.9318	0.821 (0.531–1.270)	0.3757

### Qualitative findings - the point of view of managers, physicians and nurses employed in Israeli public hospitals

The interviewees – managers, physicians and nurses, Jews and Arabs employed in public hospitals in Israel – reported requests and even demands made by Jewish (but not by Arab) patients for ethnic-based separation in inpatient rooms, which in most cases are met by the nursing staff. The interviews further reveal that in many of the cases in which nurses separate Jews and Arabs in the inpatient rooms (without being asked to do this) they do so for three reasons: to promote cultural compatibility between patients, to avoid unnecessary tension and confrontations, or because of a tendency to discriminate against Arab patients. Managers and senior physicians, on the other hand, are generally not aware of this ethnic-based separation or deny that it occurs.

#### Patients’ perspectives

Some of the interviewees, mostly the Jewish practitioners, view the public hospital as a neutral space that invites harmonious encounters between Jewish and Arab patients. As detailed below, they describe cases in which the common experience of illness blurs the otherwise distinct national and religious identities and facilitates encounters and a degree of familiarity that is not found in other domains of Israeli society. However, alongside accounts of harmonious encounters between Jewish and Arab patients, more than a few Jewish patients favor separation between the ethnic groups in inpatient rooms. Most demands for separation are made by Jewish patients and are addressed to the Jewish nursing staff. Demands for ethnic-based separation are more common at times of escalation in the Israeli-Palestinian conflict, and are notably more common among ultra-Orthodox Jewish patients.

Interviewees, particularly managers and senior physicians, describe the public healthcare system in Israel as a unique space, where Jews and Arabs can meet and even integrate:

*I always tell the story of the curtain. In the pediatric ward, an Arab and an ultra-Orthodox Jewish family shared the same room. At first, the curtain [between the beds] remained closed, and then one of the mothers had to go to the bathroom and she would ask the other mother to open the curtain a little, saying “can you look for a second?” Then, slowly, the curtain opens and they start talking (Jewish manager)*

*There are rooms where both Jews and Arabs are [placed] together. I’ve seen many beautiful examples too. An elderly Moroccan [an immigrant from Morocco] Jew, who spoke Arabic, and an Arab elder... They were in the same room, and they became good friends. It seemed to me that the Jew had no children, and the Arab’s sons took care of him (Jewish physician).*

Alongside such harmonious encounters, however, the interviewees report manifestations of hostility, tension and even racism between Jewish and Arab patients, especially during times of escalation in the Israeli-Palestinian conflict. The desire for separation felt by Jewish patients toward Arabs is clearly expressed in the former’s demands to separate them from Arab patients in the inpatient rooms. These demands are addressed to the Jewish nursing staff, and therefore appear more frequently in the responses of the Jewish interviewees.

The interviews reveal that the demands for ethic-based separation are initiated by Jewish patients, who object to being placed in the same room with Arab patients.

*The Jewish women patients want to be together in the same room. They say to me, “please, don’t put us with them” [Arab patients], they don’t even spell out with whom… I’ve never heard the opposite, that they [Arab patients] asked me not to put them with Jewish women (Jewish nurse).*

*We’ve heard this often, people asking to be moved to a “Jewish” room. They don’t want to be with Arabs… They say things that are unpleasant to hear, “I don’t want to be with these Arabs, these Arabs stink, they bring along many relatives” (Jewish physician).*

The demands of Jewish patients for ethnic-based separation occur more often during times of escalation in the Israeli-Palestinian conflict. During wartime, military operations, or terror attacks, when the atmosphere in the hospital and outside it becomes tense, there are more incidents in which Jewish patients demand to be moved to an all-Jewish inpatient room.

*It occurred mostly around the war... on a daily basis… they (Jewish patients) approached me as I was processing a new patient. They said to me, “I’m not willing to be near Arabs, I don’t want an Arab to be with me in the room” (Jewish nurse).*

The interviews indicate that demands for ethnic-based separation in the inpatient rooms are more frequently directed at the Jewish nursing staff by patients who are ultra-Orthodox Jews than by patients who are secular or traditional Jews.

*[Separating] Arab and Jewish patients is common, especially among [ultra] orthodox Jews... They may say to me, “do me a favor; I don’t want to lie next to an Arab” (Jewish nurse).*

*I began my medical career as an intern in gynecology in X (a hospital serving an ultra-Orthodox population)... There was also clear separation in the [inpatient] rooms. That is, Jewish rooms and Arab rooms within the maternity ward (Jewish physician)*

#### Nurses’ perspectives

Although from the outset we intended to focus on patients’ demands to separate Jews and Arabs in inpatient rooms, it emerged from the interviews that in many cases such separation is practiced even when patients do not request it. In other words, even though Israeli hospitals officially apply a policy of neutrality and equality in treatment, the interviews reveal that the nursing staff, including the head nurses, sometimes separates Jewish and Arab patients of their own accord and without being asked to do so. This pattern is prevalent in certain wards and not in others, and is not part of the hospital’s policy. The nurses and physicians interviewed mentioned three reasons for separating Jewish and Arab patients in the rooms: the desire to maximize cultural compatibility between patients; the aspiration to avoid unnecessary tension and confrontations in the wards and as expression of discrimination and racism on the part of staff against Arab patients.

Interviewees who note the desire for cultural compatibility between patients as a motive for ethnic-based separation between patients, speak of the practice in positive terms, describing it as a step to ensure a pleasant hospitalization experience for all patients.

*The nurses said that sometimes they really try to arrange the rooms according to group or ethnicity... [They] try, if possible, to put Arabs together. It does not always work out but in general they are aware to it... It is not a rule; “now we will turn the entire ward upside down so that these two Arabs can be together, these ultra-Orthodox [Jews] can be together, the secular patients can be together,” not at all... The consideration is cultural; to make the hospital experience more pleasant for the patient (Jewish physician).*

*Nowadays, we separate in the ward; we try to arrange different rooms for Arabs and for Jews... The rationale is that the mentality is different, the odors, the behavior (Jewish physician).*

Some nurses spoke of separation between Jewish and Arab patients not as a response to their different cultural needs but as a tactic aimed at maintaining a good atmosphere in the ward and preempting tension, problems, and complaints.

*Once there was an argument between two families in the ward. They almost killed each other. And then, when we separated them, it was all right... The Arab turns on the TV on Shabbat, the Jew comes along, shuts down the TV, and yells at the Arab… The Arabs’ ring tone is a prayer from the Koran and religious songs... It’s difficult... if it’s possible, I separate [Jewish and Arab patients]. Many nurses here, Jewish nurses, actually argue with me, “‘what are you doing?”... And they know, deep down they know, that it solves many problems (Arab nurse).*

*The policy is that we should not separate, we should not discriminate. But below the surface, I’m not sure, I can assure you that we do turn a blind eye and say okay, if there’s a chance of avoiding conflict (Jewish nurse).*

A few of the interviewees spoke about separation between Jewish and Arab patients by the nursing staff not as a matter of recognizing the importance of cultural compatibility between patients or as a means of avoiding unnecessary tension and confrontations, both of which may be construed as good intentions, but rather as a manifestation of racist and discriminatory attitudes.

*There is a large room in the ward, with six or eight beds. It’s a really terrible room... So often I’ve heard from the nurses that when they are arranging the rooms, unless there is some medical consideration, they shove the [Arab] Bedouin patients in there (Jewish physician).*

*There are certain nurses who are more racist than others, so they will separate. From the beginning, they won’t put Arabs into a room with Jewish patients (Jewish nurse(.*

Interviews reveal that ethnic-based separation within inpatient rooms occurs routinely in some maternity wards in public hospitals in Israel.

*There are maternity wards in which, contrary to every statistical distribution, there are Jewish rooms and Arab rooms, so you have to find out what the considerations were. You have to find out if there was intention to do something racist or not (Jewish physician).*

Ethnic-based separation between Jewish and Arab patients may be more prevalent in maternity wards, as interviewees explained, owing to competitive and financial considerations. In Israel, hospitals receive a generous payment for every woman who gives birth in their hospital. Moreover, women are free to choose where they wish to have their baby delivered and competition between hospitals is high.

*There is competition between hospitals, and each wants to attract more patients so everything is already linked to money, right? The private patients also bring in money, and plenty of money, to the hospital. So you don’t want to do anything that would upset them (Jewish nurse).*

Further factors that may encourage ethnic-based separation in maternity wards are the large volume of visitors and the exhaustion of the patients; and perhaps cultural differences also come to the fore when giving birth.

*It’s our tradition... When someone gives birth all the acquaintances, the entire family, all the close friends flock to the hospital to wish good luck... to congratulate. They don’t wait for her to come home and let her recover in her own good time... The entire clan, the whole neighborhood must know that she’s given birth (Arab nurse).*

#### Senior administrators’ and physicians’ perspectives

There is a striking difference between the incidence of ethnic-based separation in hospitals reported by nurses, and the denial of such incidence as reported by administrators and senior physicians. The nurses, apparently, generally comply with demands made by Jewish patients to be placed in an all-Jewish room. Only a few of them relate that they refused such a demand:

*I tell them that we arrange the rooms according to their diagnoses, according to what they need. I tell them that there are very nice [Arab] women there and you’ll meet them and you’ll find that they are good women (Jewish nurse).*

*Once a patient was brought into the ward, and I was told that because there was an Arab in the room she didn’t want to be in that room. I told her that a patient can’t return to the emergency room, so please sign a refusal form and go home... As far as treatment is concerned, we have no written guidelines saying that I should place a Jew with a Jew and an Arab with an Arab (Arab nurse).*

Given the absence of clear guidelines that prohibit ethnic-based separation in inpatient rooms, most nurses feel they cannot refuse demands for separation. Many managers and senior physicians, on the other hand, claim that there is no ethnic-based separation between Jewish and Arab patients in Israeli public hospitals. The discrepancy between the managers’ and senior physicians’ accounts of what goes on and the stories narrated by the nursing staff is notable:

*I have never heard of such a thing in the hospital. I’ve never been asked to do something like this; and had I been asked, I would not have consented to it... This is not something that could happen and not something that has happened... I’ve never heard of such a request “I do not want to be in a room with an Arab woman, I don’t want to be in a room with a Jewish woman” (Jewish manager).*

*There is no separation of rooms here, certainly not between Jews and Arabs. If patients are separated, it is probably because of gender, we do not put a man next to a woman... or if there is a particular medical reason... but on ethnic grounds?? No ... Maybe we don’t know about it, maybe they [the patients] turn to the nurses and ask them, “Can you move us into another room?” I can’t say that it never happens, but we certainly don’t not have such a policy, on the contrary (Arab manager).*

## Discussion

The survey we conducted revealed that 30% of Jews and 21% of Arabs agree that patients should be allowed to choose to be placed in an inpatient room in which only patients of their own ethnic group are hospitalized. Jews, more than Arabs (2.51 vs. 2.02, *p* < .0001) agree with this statement. A higher level of religiosity and a lower level of education predict attitudes that favor ethnic-based separation among patients, both Jewish and Arabs. In other words, Jews and Arabs who defined themselves as more religious and who are less educated were more likely to favor ethnic-based separation in inpatient rooms. While Jews, more than Arabs, agree with separation in inpatients rooms, the survey furthermore revealed that most of the respondents, both Jews and Arabs (1.68 vs. 2.04, ns), opposed the division of the entire healthcare system into two separate systems – one for Jews and another for Arabs, in line with the de facto separation between most Jewish and Arab students practiced in Israel’s education system.

The interviews conducted with both Jewish and Arab managers, physicians and nurses employed in public hospitals in Israel indicate that demands and requests are made by some Jewish (but not Arab) patients to be separated in the inpatient rooms, which are in most cases met by the nursing staff. The use of both the terms “requests” and “demands” by the interviewees points to the relative power wielded by the practitioners interviewed and their subjective experiences at work. Apparently, practitioners who feel able to refuse to separate the patients tend to perceive their approach as a request, while practitioners who feel unable to deny the patients’ wishes experience their approach as a demand. These demands crop up more frequently at times of escalation in the Israeli-Palestinian conflict, and were more often initiated by ultra-orthodox Jewish patients than by patients who are secular or traditional Jews.

While the survey we conducted revealed that a considerable minority of both Jews and Arabs support ethnic-based separation in inpatient rooms, the practitioners we interviewed, and particularly the nurses, mentioned only demands for separation made by some Jewish patients. This gap may be related to the relatively small number of interviewees (50) compared to the survey. Alternatively, it may be that Arabs, as members of a minority population, do not expect to be placed exclusively with other Arab patients. Furthermore, it may be that even if some Arabs may prefer such a separation, given the power relations that pertain in Israeli society, public hospitals included, they rarely demand or request it.

It is the nurses who are in charge of placing patients in rooms, and it is therefore they who have to handle the demands/requests made by some Jewish patients to be separated from Arab patients. The interviews reveal that, in certain wards, the nurses sometimes separate Jewish and Arab patients of their own accord, before they are asked to. In other words, in some wards the nurses implement an informal policy of occasional separation, which remains covert and unarticulated and runs contrary to the ethos of neutrality in medicine. The nurses raised three reasons for acting in this way: an aspiration to achieve cultural compatibility between patients and provide a pleasant hospital experience for patients who are placed in rooms with people who are similar to them; the desire to maintain an air of tranquility and avoid unnecessary tension and confrontations; and at times, manifestations of racism and discrimination against Arab minority patients. From the interviews we cannot, however, assess what proportion of the separation is implemented in order to promote cultural compatibility and avoid confrontation, and how much of it is linked to discrimination.

While the nurses may comply with the Jewish patients’ demands for ethnic-based separation in the inpatient rooms, the managers and senior physicians interviewed deny that this form of separation is practiced in the hospital wards. Perhaps they are not aware that separation is practiced, since the patients’ demands are handled by the nurses and are not referred to them; or perhaps, as the official representatives of the hospitals who bear legal responsibility for its ethical functioning, they are reluctant to confirm explicitly the existence of what they know to be an ethically problematic phenomenon. In practice, it is precisely their status, authority and prestige that enable them to refuse demands made by Jewish patients to be placed in a room with other Jews. Yet they take a back seat and leave the nurses to handle these situations, lacking clear guidelines or a formal policy.

## Conclusions

We recommend publishing and enforcing a clear explicit policy prohibiting ethnic-based separation of patients in Israeli public healthcare system. Such a policy would help the nursing staff to cope with demands/requests made by Jewish patients to separate them from Arab patients, and would perhaps deter patients from making these demands/requests altogether. In addition, we recommend enforcing the restrictions on the number of visitors in the wards, especially in the maternity ward, and allowing multiple visitors (apart from spouses that may accompany the mothers freely), only in visiting hours and out of the inpatients rooms, since the interviews reveal that the issue of multiple visitors often underlies the demand to separate Jews from Arabs in the inpatient rooms, whether this be a genuine concern or an excuse. Emphasis should be placed on implementing this policy in hospitals that serve high concentrations of very religious populations.

Future research can compare the attitudes of patients who belong to different streams of Judaism, and those who belong to different Arab groupings (e.g., Muslins and Christians) regarding the subject. It would also be useful to assess the extent to which, in practice, Arab and Jewish patients are accommodated within the same rooms in various departments and hospitals, by conducting random, unannounced hospital inspections.

In order to enforce the policy, the Ministry of Health should raise awareness of the issue among healthcare practitioners. In practice, as the interviews show, some staff members, particularly nurses, fail to understand why ethnic-based separation is unacceptable, and in fact condone those patients who demand it, either on racial or cultural grounds. It is important to impress upon them that the way to harmony and to preventing descent into ethnic segregation runs through inter-ethnic encounter, dialogue and acquaintance, rather than through separation.

Longitudinal studies suggest that the causal link between contact and prejudice is bidirectional – prejudice reduces contact but contact also reduces prejudice [4, 7]. Research on intergroup contact has mostly viewed desegregation as a necessary condition for contact to unfold its power to reduce prejudice, promote intergroup harmony and create a more tolerant society [9, 22]. It is therefore important to prevent separation from becoming a standard and institutionalized practice among nursing staff, which might lead to formal segregation throughout hospitals.

## Footnotes

1 The term ‘Arabs’ is used throughout the paper. Although many Israeli Arabs consider themselves part of the Palestinian people, the term ‘Arabs’ rather than ‘Palestinians’ is used here to denote Arab minority Israeli citizens, and in order to avoid confusion with Palestinian residents of the Palestinian Authority.
